# Challenges in the introduction of next-generation sequencing (NGS) for diagnostics of myeloid malignancies into clinical routine use

**DOI:** 10.1038/s41408-018-0148-6

**Published:** 2018-11-12

**Authors:** Ulrike Bacher, Evgenii Shumilov, Johanna Flach, Naomi Porret, Raphael Joncourt, Gertrud Wiedemann, Martin Fiedler, Urban Novak, Ursula Amstutz, Thomas Pabst

**Affiliations:** 1 Department of Hematology and Central Hematology Laboratory, Inselspital, Bern University Hospital, University of Bern, Bern, Switzerland; 2 Center for Laboratory Medicine (ZLM)/University Institute of Clinical Chemistry, Inselspital, Bern University Hospital, University of Bern, Bern, Switzerland; 3 Department of Hematology and Medical Oncology, University Medicine Göttingen (UMG), Göttingen, Germany; 4 Department of Hematology and Oncology, Medical Faculty Mannheim of the Heidelberg University, Mannheim, Germany; 5 Department of Medical Oncology, Inselspital, Bern University Hospital, University of Bern, Bern, Switzerland

## Abstract

Given the vast phenotypic and genetic heterogeneity of acute and chronic myeloid malignancies, hematologists have eagerly awaited the introduction of next-generation sequencing (NGS) into the routine diagnostic armamentarium to enable a more differentiated disease classification, risk stratification, and improved therapeutic decisions. At present, an increasing number of hematologic laboratories are in the process of integrating NGS procedures into the diagnostic algorithms of patients with acute myeloid leukemia (AML), myelodysplastic syndromes (MDS), and myeloproliferative neoplasms (MPNs). Inevitably accompanying such developments, physicians and molecular biologists are facing unexpected challenges regarding the interpretation and implementation of molecular genetic results derived from NGS in myeloid malignancies. This article summarizes typical challenges that may arise in the context of NGS-based analyses at diagnosis and during follow-up of myeloid malignancies.

## Biological-clinical challenges

### Defining the clinical impact of novel NGS markers for different myeloid entities

Current diagnostics of myeloid malignancies, including acute myeloid leukemia (AML), myelodysplastic syndromes (MDS) and myeloproliferative neoplasms (MPN) has been rapidly evolving^1,2^. Within the last 5–10 years, next-generation sequencing (NGS) has been introduced in most specialized hematologic laboratories with various myeloid NGS panels now being commercially available. These panels are based on targeted resequencing and usually analyze 25–50 genes. The genes tested within these panels can be classified into several functional categories including the splicing machinery (e.g., *U2AF1, SF3B1, SRSF2, ZRSR2*), epigenetic modifiers (such as *TET2, DNMT3A, BCOR, ASXL1, IDH1, IDH2*), cohesins (*STAG2, RAD21, and SMC3*), transcription factors (*RUNX1, WT1, ETV6*), signaling molecules (*NF1, NRAS, CBL, PTPN11, JAK2, FLT3*), and chromatin modifiers (*EZH2, ASXL1*)^3–6^.

**Table 1:** Challenges accompanying the introduction of massive parallel sequencing in clinical routine diagnostics in hemato-oncology

Challenge	Background	Current and future approach
Discrimination of leukemia-related mutations from polymorphisms or passenger mutations	Driver mutations expected to occur at higher allele frequency in patient samples than passenger mutations; driver mutations more likely to have an impact on protein function than polymorphisms or passenger mutations	Optimization of cancer-specific databases including reporting of rare physiological gene variants Implementation of novel bioinformatic algorithms based on prediction of functional impact Quantitative and dynamic VAF monitoring (separately and together with other mutations) at follow-up
Discrimination of somatic leukemia-related mutations from CHIP	CHIP is presented in ~10% of individuals aged 70 to 80 and in up to 20% in the age group > 80 years	Quantitative and dynamic VAF monitoring (separately and together with other mutations) at follow-up Clarifying the significance of CHIP in the context of myeloid malignancies
Discrimination of leukemia-related somatic mutations from pathogenic germline alterations	Challenge to differentiate acquired somatic mutations from germline pathogenic variants at diagnosis	Mutation detection in germline control samples (e.g., skin fibroblasts, saliva) in mutations such as in *RUNX1*, *CEBPA* Thorough medical family history followed by molecular genetic tests in relatives if necessary High and stable VAF (e.g., 40–50%) at follow-up despite clinical response to treatment may be indicative for germline alteration
Discrimination of true genetic alterations from PCR, sequencing and post-sequencing artifacts	Many artefacts are known to arise during NGS library preparation, sequencing and data analysis	Error correction using molecular identifiers that individually label original input DNA molecules Refinement of error-correction computational methods in post-sequencing NGS data analysis Confirmation using Sanger sequencing
Limited sensitivity of NGS for minimal residual disease (MRD) assessment	Mutations detected at diagnosis may be re-identified at best to a VAF of 1–2%	Error-corrected sequencing using molecular identifiers Complementation of NGS by established MRD tools like real-time PCR and flow cytometry
High financial burden; demand on interdisciplinary approaches	Expensive technical and staff equipment, sophisticated data interpretation Complex translation of NGS results into therapeutic decisions	Development of continuously updated NGS interpretation sets and algorithms for well-established mutational profiles within distinct hematological malignancies Interdisciplinary leukemia boards

*VAF* variant allele frequency, *CHIP* clonal hematopoiesis of indeterminate significance, *bp* base pairs, *G* guanine, *C* cytosine, *ITDs* internal tandem duplication

With the help of myeloid gene panels more than one recurrent somatic mutation can be identified in most AML patients, and even within defined AML entities additional molecular genetic mutations are detectable in many cases^7^. In MDS, NGS allows the identification of molecular mutations in nearly 90% of the patients^3,5,8–11^. As a result, molecular genetic markers find increasing entrance into current classification systems. In this context, the favorable subcategory of MDS with ring sideroblasts has been expanded to include cases with ≥5% of ring sideroblasts in the setting where a *SF3B1* mutation is present in the most recent revision of the WHO classification^12^. Also for AML, new molecularly-defined entities have been suggested including chromatin–spliceosome, *TP53* aneuploidy, and provisionally, *IDH2*R172^4^.

The molecular genetic landscape undergoes exploration by NGS also in *BCR-ABL1*-negative MPNs^13–16^. Besides the three classical mutations in *JAK2*, *CALR*, and MPL that are commonly referred to as MPN driver mutations, non-driver mutations in the genes known from MDS and AML are also detected in polycythemia vera (PV), essential thrombocythemia (ET), and primary myelofibrosis (PMF)^15,17,18^. However, as compared to AML and MDS, the clinical significance of these novel markers has been more difficult to define. Considering the importance of cytomorphology and histopathology in addition to the high frequency of the three driver mutations in the MPNs, which may also be investigated by traditional molecular techniques^12^, the value of NGS at present lays in the refinement of risk stratification in critical or difficult cases and consequently treatment decisions^19,20^. Along this line, the prognostic impact of certain co-occurring mutations has been associated with MPN disease progression as well as the development of secondary AML^21^. This is particularly relevant in PMF, where high molecular risk (HMR) markers include mutations in *ASXL1, SRSF2, EZH2, IDH1* and *IDH2*^18^. Similarly, in ET and PV, mutations with adverse prognostic value are *IDH2*, *U2AF1*, *EZH2*, *TP53*, *SH2B3*, and *SF3B1*^14^. The number of co-occurring mutations has also been suggested to be prognostically relevant with ≥2 HMR mutations predicting the worst prognosis and shortened leukemia-free survival^22^. Thus, NGS enables the identification of patients that are at higher risk for progression and transformation, whereas during follow-up, the occurrence of clonal molecular evolution or the detection of HMR marker can identify MPN patients that may become potential candidates for allogeneic hematopoietic stem cell transplantation (HSCT)^23^.

Besides allowing a refinement of risk stratification and therapeutic decision making in patients with driver mutations, NGS offers great benefit in about 10% of patients with pathologic features of MPN that lack defining molecular drivers (i.e., *JAK2*V617F, *CALR*, and *MPL*)^14,24^. Diagnostics in these so-called triple-negative (TN) cases can be challenging, so NGS may confirm the presence of hematological clonal disease and corroborate an initial cytomorphologic diagnosis. TN patients may carry driver mutations in non-canonical sites in *JAK2* and *MPL* or in alternative genes including epigenetic modifiers (*ASXL1, TET2*), the spliceosome (*SF3B1, SRSF2*), and regulators of cytokine signaling (*CBL, SH2B3*)^14^. However, despite growing implementation of myeloid NGS panels, a small proportion of TN MPN patients remains without any mutation detectable.

Together, the growing insights into molecular aspects of the pathogenesis of myeloid malignancies will eventually pave the way towards a more detailed clinical evaluation and optimized therapeutic decisions.

### Discriminating leukemia-related mutations from genetic polymorphisms and passenger mutations

Following the expansion of large-scale cancer genome sequencing, databases such as Genome Aggregation Database (gnomAD), 1000-Genomes-Projekt and Exome Aggregation Consortium aim to depict population allele frequencies in detail^25–27^ (Table 1). Other databases (Table 2A), e.g., International Agency for Research on Cancer, Catalog of Somatic Mutations in Cancer, Cancer Genome Atlas, and Human Gene Mutation Database focus on somatic and germline mutations to distinguish particular gene variants between “true” tumor associated mutations and genetic polymorphisms^28–32^. Some of these databases are accessible via genome browser apps (e.g., Alamut). Based on such tools, gene variations described with frequencies of more than 1% in the population represent genetic polymorphisms.

**Table 2A:** Overview on databases used for the characterization of genetic variants

Database	URL	Description
cBioPortal	www.cbioportal.org	Free database for cancer genomics
My Cancer Genome	www.mycancergenome.org	Free database for cancer genomics
COSMIC	cancer.sanger.ac.uk/cosmic	Catalogue Of Somatic Mutations In Cancer, free database for somatic mutations in cancer
dbSNP	www.ncbi.nlm.nih.gov/SNP	Free database for short genetic variations
ExAC Browser	exac.broadinstitute.org	Freely available exome sequencing data from the Exome Aggregation Consortium
ClinVar	www.ncbi.nlm.nih.gov/clinvar	Free database for information about genomic variations and their relationship to human health
gnomAD	gnomad.broadinstitute.org	Genome Aggregation Database, free database for genome sequencing data
ESP	evs.gs.washington.edu/EVS	Exome Sequencing Project, free database for exome sequencing data
LOVD	www.lovd.nl	Leiden Open Variation Database, freely available tool for gene-centered collection and display of DNA variations
HGMD Professional	www.hgmd.cf.ac.uk	Database for known gene mutations causing inherited diseases

In sight of the enormous number and variety of gene alterations detectable by NGS, the discrimination of leukemia-initiating mutations from incidental passenger mutations lacking any impact on leukemogenesis can be challenging. In fact, only a small fraction of somatic gene alterations seems to act as driver mutations that are involved in cancer initiation and progression^33,34^. Different bioinformatic methods are utile to discriminate driver mutations from passenger variants, either by assessing the frequency of mutations or by predicting their functional impact^35,36^. The first approach assumes a driver gene to be mutated in a significantly higher proportion of alleles compared to the expected background mutational rate and is calculated based on statistical scores (e.g., CancerMutationAnalysis, CHASM, DMI) taking into account, amongst others, gene size, nucleotide constitution and background non-synonymous mutation rates from cancer-specific databases (e.g., COSMIC)^37–39^. The second approach is based on the hypothesis that mutations with “damaging” functional impact, predicted by tools like SIFT, MutationAssessor or MAPP, are more likely to represent driver mutations than those with little (if any) predicted impact^35,36^. (Table 2B)

**Table 2B:** Overview on algorithms used for the validation and interpretation of genetic variants

Algorithm	URL	Description
Align GVGD	agvgd.hci.utah.edu	Free web-based program for classification of missense variants
SIFT	sift.bii.a-star.edu.sg	Free web-based program for prediction of missense variant effect on protein function
MutationTaster	www.mutationtaster.org	Free web-based program for prediction of disease-causing potential of DNA variants
PolyPhen-2	genetics.bwh.harvard.edu	Free web-based program for prediction of missense variant effect on protein structure and function
Provean	provean.jcvi.org	Free web-based program for prediction of missense and indel variant effect on protein function
FATHMM	fathmm.biocompute.org.uk	Free web-based program for prediction of functional consequences of coding and non-coding variants

To further illustrate the difficulties that may arise in the discrimination of germline polymorphisms from “true” somatic mutations, we detected a c.167 T > C, p.(Leu56Ser) alteration in the *RUNX1* gene in a patient with therapy-related AML. In the PB, the variant allele frequency (VAF) was 44% at diagnosis. At 9 months from diagnosis, the patient was in remission, however, the respective *RUNX1* alteration persisted at a VAF of 48% in the PB, whereas a co-incidental *SF3B1* mutation had decreased from 10.5 to 2.9% in the PB. At this later time point, we identified the aforementioned *RUNX1* alteration as a germline polymorphism occurring at a frequency of around 1.5% in the general population (gnomAD). This information had not been available at diagnosis of the AML. Thus, considering the steadily improvement of molecular genetic databases, population frequency data are expected to become more and more available even for very rare germline variants in the near future.

Nevertheless, the evidence underlying the respective databases is not without limitations and a satisfying definition of levels of evidence is lacking. Browsers that facilitate data aggregation and display such as Alamut require further optimization regarding these functions. Thus, the review of the reported genetic variants by experts with technical and clinical knowledge is highly relevant, and literature research remains mandatory, too. Interdisciplinary teams of expert physicians, genetic counselors, and technologists are required to improve the level of accuracy of genomic databases and the interpretation of distinct genetic variants that may occur in myeloid malignancies. In addition, further high quality clinical trials and functional studies are needed to improve our understanding of the relevance of less well-characterized genes and gene mutations.

### Discrimination of somatic leukemia-related mutations from CHIP

The discrimination of somatic leukemia-associated mutations from clonal hematopoiesis of indeterminate potential (CHIP) is another challenging issue^40,41^. Clonal hematopoiesis is typically benign in healthy individuals with very small clones, while patients with clinically abnormal hematopoiesis, larger clones and more driver gene mutations appear to be at much greater risk^42^. CHIP is defined by evidence of a somatic mutation in a leukemia-associated driver gene amounting to an allele frequency of 2% or more in individuals that do not fulfill the WHO criteria for a hematologic malignancy^43^. Kwok et al. identified somatic mutations in myeloid malignancy genes in 71% of patients with MDS, 62% of patients with ICUS (idiopathic cytopenia of unclear significance) and evidence of some dysplasia, and in 20% of ICUS patients without dysplasia. Variant allele fractions were comparable between patients with clonal ICUS and MDS^44^.

In AML, *DNMT3A* mutations can persist in the post-therapeutic period despite continuous remission without affecting the relapse rate in the absence of co-incidental gene mutations^45,46^. Aside from *DNMT3A*, the spectrum of CHIP may comprise a wide range of genes including *TET2*, *ASXL1*, *RUNX1*, *IDH1*/*2*, and others. Jongen-Lavrencic et al. recently demonstrated no adverse effect of persisting *DNMT3A*, *TET2* and *ASXL1* mutations in the absence of other genetic alterations in a cohort of 482 AML patients achieving first remission after two cycles of intensive induction treatment, thereby allocating them to CHIP rather than to a pre-relapse condition. Yet, any additional mutation present in the same patients with mutated *DNMT3A*, *TET2*, and *ASXL1* had a dramatic impact on the cumulative relapse incidence^47^. Following this line of research, we recently reported a patient with *NPM1* mutation subtype switch at relapse of AML 8 years after successful intensive chemotherapy and consolidation with autologous stem cell transplantation (*NPM1*mut subtype D at first diagnosis; subtype A at relapse). Interestingly, at every assessment since diagnosis, a *DNMT3A* mutation was present at allele fractions between 37 and 51%^48^. Furthermore, the *JAK2*V617 mutation, which constitutes one of the most common mutations described in CHIP, occurs in about 0.1% of the general population without clinical signs of myeloproliferative disease^49–51^. Therefore, caution should be used in order not to refer a patient automatically to a diagnosis of MPN in the case of a *JAK2* mutation detected by traditional molecular techniques or NGS. This aspect emphasizes the need to clearly separate true MPNs from CHIP when a *JAK2* mutation has been detected, e.g., by considering the results of a bone marrow biopsy. Clearly, the role of clonal hematopoiesis requires careful investigation considering the long-term courses of patients. In addition, patients with unexplained thrombocytosis should not automatically be assigned to a diagnosis of MPN in case of detection of a mutation such as *DNMT3A* or *TET2* (that may occur in myeloid malignancies but may also reflect clonal hematopoiesis).

Naturally, while the differentiation between CHIP and leukemia-associated mutations is important, the finding of CHIP itself deserves attention as well. Allogeneic stem cell donation from individuals harboring CHIP, e.g., of *DNMT3A* may result in poor hematopoietic engraftment in the allograft recipients^52^. Individuals with CHIP have an increased risk for various myeloid malignancies as well as for cardiovascular disease due to accelerated arteriosclerosis, probably secondary to vascular inflammation driven by clonally derived monocytes/macrophages^43^.

### Discrimination of leukemia-related somatic mutations from rare pathogenic germline alterations

Considering the variety of mutations such as *TP53*, *RUNX1*, *GATA2*, *CEBPA*, or *ASXL1* that may arise in the context of AML or MDS, but may also occur as rare pathogenic germline variants, there is considerable risk for misinterpretation^53–55^. The “IARC TP53 Database” contains more than several hundred *TP53* germline mutations, most of which are associated with the Li-Fraumeni cancer predisposition syndrome, do not show any hotspots regions and often occur within exons 2–11^56,57^. Recently, germline *TP53* mutations were reported in 6 out of 107 patients with treatment-related AML highlighting their role in leukemogenesis after cancer treatment^58^. Up to 11% of AML patients with biallelic *CEBPA* mutations, in fact, harbor a mutant *CEBPA* germline predisposition. In contrast to somatic *CEBPA* mutations, which cluster in the C-terminus of the gene, germline mutations mostly affect the N-terminus^55,59,60^. *RUNX1* germline mutations are associated with familial platelet disorder with predisposition to AML, they mostly occur in a mono-allelic form and - in contrast to sporadic AML - represent the initiating molecular genetic event in leukemogenesis^61^. Thus, the search for a potential germline origin of the mutation may be justified when NGS detects an isolated *RUNX1* alteration with a mutation load around 50% in a given AML patient. Following the increasing implementation of NGS in routine cancer diagnostics, a higher frequency of germline mutations will be detectable in the future. Along this line, Drazer et al. identified pathogenic or likely pathogenic variants in genes associated with hereditary hematopoietic malignancies in 21% of 360 patients with hematologic malignancies (*ANKRD26*, *CEBPA*, *DDX41, ETV6, GATA2, RUNX1* and *TP53*). In addition, they were able to show that especially mutations with VAF of more than 40% were more likely to be germline mutations as demonstrated by parallel germline tissue analyses^62^.

In the MPNs, complexity is added by the recent identification of a number of rare germline predisposition alleles^49,63^. Affected genes include *TERT, SH2B3, TET2, ATM, CHEK2, PINT, FG11B*, *MECOM, TERT, JAK2 and HBSL1-MYB*, respectively^49,63^. The prognostic value of these heritable genetic polymorphisms is not fully understood and is thus not currently recommended be used for estimating the risk of developing an MPN. As NGS is unable to distinguish between germline and somatic origins unless paired germline/tumor DNA samples are evaluated the individual family’s history needs to be carefully taken into account^24^. Similarly, driver MPN mutations may also be present in rare forms of hereditary thrombocytosis or erythrocytosis^64,65^, where cytoreductive therapy is not currently recommended^24^.

In clinical practice, true germline control samples derived from non-hematopoietic, non-malignant tissues like skin biopsies are not always available. The examination of saliva provides an alternative (albeit with a lower safety level due to the possible contamination by hematopoietic cells). In many cases, and when population frequency databases do not provide definite information, only follow-up monitoring of a given mutation from the bone marrow or PB allows clarification. When a patient achieves complete remission, but the respective mutation persists at the previously high allele fraction, an interpretation as CHIP or germline mutation (especially in the case of a mutation load around 50%) seems more likely. In cases where different mutations occur at the primary diagnosis and all other mutations decrease under therapy, a single persisting mutation requires diligent re-evaluation. In such cases, the analysis of non-myeloid tissue is mandatory for exclusion of a hereditary origin of the respective alteration.

### Detection of reciprocal rearrangements

Commercial myeloid NGS panels nowadays also enable the detection of reciprocal gene rearrangements. In the pre-NGS era, a limited number of reciprocal rearrangements underwent screening by PCR at diagnosis of AML, such as *PML-RARA*, *RUNX1-RUNX1T1*, and *CBFB-MYH11*. In addition to these frequent rearrangements (that are usually included in myeloid panels), current NGS panels allow the detection of a broad variety of rare reciprocal rearrangements in hemato-oncologic malignancies^66,67^. In fact, over the past years more than 9000 new fusion genes - mostly interpreted as passenger events by comparison with the data of the CancerGenome Atlas network - have emerged following the introduction of NGS^68^. This reflects the increased sensitivity of NGS to discover subtle intra-chromosomal rearrangements whereas fluorescence in situ hybridization (FISH) may only detect exchanges of considerably larger chromosome segments. Accordingly, 75% of gene fusions revealed by NGS are related to intra-chromosomal rearrangements^68^.

Stengel et al. investigated targeted RNA sequencing by NGS for the detection of reciprocal rearrangements in 58 AML cases with suspected novel fusions based on the detection of only one partner gene (*RUNX1, ETV6, PDGFRB, KMT2A, RARA, NPM1, MECOM, PDGFRA, BCOR, TET2, NUP98*) by chromosome banding analysis and FISH. The second partner gene could be identified in 59% of the patients by extended RNA sequencing^69^. Another example are unusual variants of APL with pathogenic *X*-*RARA* or alternative *PML-RARA* fusions revealed by NGS, which traditional methods including RT-PCR failed to detect^70^. In a patient with t-AML and a complex aberrant karyotype including a trisomy 8, we recently detected a cryptic reciprocal *RUNX1-CBFA2T3* rearrangement [corresponding to t(16;21)(q24;q22)] in a total of 25,000 NGS reads. Metaphase FISH using a *RUNX1* break apart probe confirmed the t(16;21). According to the literature, t(16;21) positive AML is frequently associated with previous radio-/chemotherapy, typically demonstrates additional chromosomal aberrations including trisomy 8, and patients may show eosinophilia^71^.

In cases with high transcript numbers, the correct diagnosis is easy to obtain, whereas low transcript numbers render it difficult to discriminate true genetic alterations from artifacts or passenger mutations according to the above-described principles of driver gene detection. Interphase FISH can confirm cryptic rearrangements below the detection level of chromosome banding analysis, if commercial probes for the involved genes/breakpoints are available. The combination with FISH allows excluding false positive results in the case of higher read counts. On the other hand, the addition of FISH increases the costs and the turn-around-time. The clinical impact of rare fusion transcripts detected only by FISH but not by chromosome banding deserves diligent investigation in the next years.

## Technical challenges

### Discrimination of true genetic alterations from PCR, sequencing, and post-sequencing artifacts

NGS procedures are associated with sequence errors due to artifacts that originate from library preparation, the sequencing process itself, or data analysis (e.g., read mapping, variant calling) resulting in incorrect calling of DNA bases or sequence variants^72–74^. Such challenges may result from the investigated genomic region itself with under- or overrepresentation of particular amplicons due to sequence-specific biases in target enrichment or sequencing efficiency (Table 1). Indeed, the depth of coverage across the target region can differ between library preparation methods, with amplicon-based protocols overall showing less uniform coverage compared to hybridization-capture-based target enrichment^75^. In addition, distinct sets of nucleotides can be associated with poor sequencing performance (e.g., GGT or GGC patterns for Illumina technique or homopolymer regions for Ion Torrent)^76^. Thus, NGS quality depends on the properties of the target sequence^77–80^. However, sequence differences in NGS reads may also reflect PCR errors such as base misincorporations or rearrangements occurring in the process of massive and simultaneous amplification within multiple rounds of PCR^81,82^. With the increasing use of molecular identifiers (i.e., molecular barcodes that individually label each DNA molecule from the original sample), artifacts arising both from PCR amplification or sequencing may be significantly reduced due to the use of consensus sequencing of single DNA molecules^83^.

Incorrectly called sequence variants may also arise from digital processing during data analysis. This phase, also called post-sequencing NGS pipeline, represents a multistep and sequential process including quality control of raw sequence reads; aligning to a reference genome/assembly; post-alignment quality control and recalibration; identification of mutations (variant calling and genotyping); post-variant call/genotyping quality control; and finally data storage^77,84,85^. Although all these processes include some error-correction procedures, further computational methods are necessary to reduce the sequential bias at this stage.

Besides further improvement of NGS-based assays and bioinformatic pipelines, additional Sanger sequencing may be helpful to discriminate errors caused by NGS from true genetic alterations. However, the limited sensitivity of Sanger sequencing of around 20% does not allow the investigation of sequence alterations detected by NGS at lower allele fractions occurring e.g., in MDS or AML subclones. Even with further improvement of NGS-based assays and bioinformatic pipelines, overdependence on bioinformatics pipelines should be avoided. For example, indel mutations can be incorrectly called. Visual inspection of the aligned sequencing data is advisable in cases where there is a suspicion of incorrect mutation calling by the used tool^86^. Additionally, Sanger sequencing may be helpful to discriminate errors caused by NGS from true genetic alterations.

In this context, consideration needs to be given to the impact of the reference sequences used on read mapping, variant calling and annotation. In particular, variant annotation may differ depending on the transcript set used^87,88^. In addition, newer versions of the human reference genome (GRCh38) are incorporating additional genomic regions and are increasingly accounting for population variation through the inclusion of alternate haplotypes in order to reduce reference sequence bias in read mapping^89^. Changes in the reference sequence may thus require re-validation of performance characteristics of NGS methods. This explains, for example, why GRCh37 is still in use, although GRCh38 has been published some time ago. Furthermore, future challenges include the incorporation of alternate haplotypes into variant calling algorithms^89^.

### Other technical aspects

The currently used NGS technologies in clinical diagnostics usually are classifiable as short read sequencing (read length ~100–~500 bp). Short read sequencing is inherently prone to miss structural variants such as longer insertions and deletions. This holds true especially for capture based target enrichment as opposed to targeted library amplification methods as there may be insufficient positioning data to align the obtained reads to the reference correctly^90–92^. Paired end sequencing can mitigate this issue by allowing sequencing of the same read from both ends providing additional positioning information compared with single end sequencing^90,93^. This method of sequencing comes at a cost of more complex library preparation and data analysis, higher sequencing times and data volumes^90–92^. With the ongoing maturation and future establishment of long read sequencing technologies in clinical diagnostics further tools will be available to complement the current short read sequencing applications. Long read sequencing can generate read lengths exceeding several kilobases. As such, it is much better suited to analyze structural variants and highly repetitive sequences. Additionally, it will much more readily allow direct phasing of concurrent genetic variants (cis/trans). Currently, however, the high costs of such sequencers still largely limits them to research settings^90–92^.

### Minimal residual disease

The multitude of molecular markers in AML detectable by standard myeloid NGS panels (e.g., *RUNX1*, *EZH2*, or spliceosome mutations), offers the possibility of performing molecular minimal residual disease (MRD) monitoring in virtually every patient. Most AML patients show more than one molecular marker^4^. However, MRD detection is limited by the background noise of NGS^94,95^. Already known mutations are re-identifiable at best at a variant allele fraction as low as 1%. Thus, the sensitivity of MRD detection by NGS is limited compared to quantitative real-time PCR (qPCR) offering sensitivities of 10^−4^ to 10^−6^, or the sometimes even more sensitive digital droplet PCR (ddPCR). Currently, qPCR or ddPCR are primarily used for monitoring of frequently occurring genetic alterations, e.g., hotspot mutations such as *NPM1* subtype A, or recurrent reciprocal rearrangements such as *RUNX1-RUNX1T1*/t(8;21). Therefore, increasing the number of genes within a molecular MRD marker panel is of paramount importance. An additional challenge is the frequently polyclonal character of AML, as different clones may respond differently to therapy or may re-emerge separately in the post-therapeutic or post-transplant period. Error-corrected or barcoded sequencing using molecular identifiers increasing the sensitivity of NGS to around 10^−5^ may facilitate future MRD strategies^96^. At present, a combination, e.g., of qPCR/ddPCR and NGS in the case of suitable marker combinations should be explored^47^. Given the variety of the molecular markers and their combinations, evaluation of molecular MRD results often remains individualized and requires a close interaction with clinicians. Similarly, molecular MRD results should be correlated with peripheral blood values and the results of flow cytometry, cytomorphology, and histopathology, eventually also with cytogenetics/FISH.

When AML patients develop an overt or molecular relapse, clinicians should be alert to initiate comprehensive mutation screening again. This allows for the detection of therapeutic targets such as *IDH1*, *IDH2*, or *FLT3*-ITD/TKD that may have newly developed due to clonal evolution.

### Economic and organizational aspects

Finally, the additional financial burden by the use of NGS deserves some consideration. Expenses concerning technical and staff equipment, including a variety of professionals such as molecular and computational biologists, genetic counsellors, and specialized clinicians, are considerably higher than anticipated some years ago. Even though the costs of a single NGS assay will continue to decrease, the number of genes that are required for comprehensive diagnostic results and treatment decisions is continuously growing. Therefore, the time allocation for the post-sequencing steps such as filtering of variants, comparison of data with genomic databases, and interpretation of gene variants as well as writing of laboratory reports has become a challenge. In addition, high in-depth knowledge and experience is required to meet all the demands of NGS procedures used in hematology with its extensive gene/hot-spot panels for the different hematologic subtypes.

At present, therapeutic concepts focus on allocating AML patients at the earliest possible time point to targeted therapies such as IDH1, IDH2, or FLT3 inhibition. Considering the long turnaround time of NGS, this is a relevant challenge for molecular laboratories. Thus, in many instances, molecular laboratories perform traditional assays (e.g., fragment analysis for *FLT3*-ITD or –TKD) in parallel to NGS, even though NGS provides the same results a few days later. This further increases the efforts and the costs for molecular analyses in leukemia patients. “Best-practice algorithms” that take labor and equipment resources into account and that aim to avoid duplicate analyses are needed.

### Interdisciplinary approaches

For a correct interpretation of the variety of NGS results in the context of hematological diagnostics, a constant bidirectional interaction between laboratory scientists, technicians and clinicians is essential. This holds true for the selection of the appropriate NGS panels at diagnosis, the selection of markers for follow-up analyses in accordance to the suspected hematological malignancy, but also to the interpretation of the genetic alterations detected by modern sequencing approaches. Specifically, the results of NGS should be interpreted in the context of other laboratory findings regarding cytomorphology, histopathology, immunophenotyping, traditional molecular genetics, cytogenetics, and clinical diagnostic and data outcome. NGS-based MRD approaches in principle are more comprehensive in light of the variety of mutations detectable at AML diagnosis today, but limited resources require a diligent selection of the optimal time points and the markers for NGS follow-up and a comparison with traditional and currently more sensitive approaches (e.g., real-time PCR). Thus, informal and formal interdisciplinary communication (i.e., in the context of leukemia boards, molecular tumor boards) has shown to be particularly useful.

## Conclusions

NGS has opened new horizons for individualized diagnostics and therapy of myeloid malignancies. While new technological advances may improve the sensitivity and accuracy of NGS-based analyses, its results also deserve cautious interpretation considering the clinical context. In particular, the clinical value of NGS needs to be defined for the different myeloid entities. For AML, the additional value of comprehensive analysis by NGS has to be compared to the previously limited molecular panels including some driver mutations only, such as *NPM1* or *FLT3*-ITD. The identification of additional prognostically relevant mutations such as the adverse *RUNX1* mutations or the identification of novel therapeutic targets such as *IDH1* or *IDH2* justify the additional demand that arises from comprehensive NGS analysis^97^. For MDS patients, molecular analysis allows a more accurate prognostication as compared to cytogenetics alone^5^. Nevertheless, the WHO classification of myelodysplastic syndromes so far only respects the *SFB3B1* mutation^12^ due to the overlap of MDS-related mutations with CHIP. In the MPNs, NGS can confirm a clonal disorder in triple-negative cases and improves the characterization of the risk profile, e.g., in patients with PMF. Presence of two or more high molecular risk markers such as mutations in *ASXL1, EZH2*, or *IDH1*/*IDH2* predicts a highly adverse prognosis and rapid leukemic transformation^22^ and thus may identify potential candidates for allogeneic HSCT^23^.

Apparent AML or MDS-associated mutations may reflect germline variants or clonal hematopoiesis. In critical cases, exclusion of a germline origin by analysis of buccal cells, or, ideally, a skin biopsy, is mandatory. Persistence of mutations such as *ASXL1*, *DNMT3A*, or *TET2* despite clearance of other mutations in AML patients under therapy should raise alertness for the possibility of clonal hematopoiesis^47^. Molecular MRD diagnostics is likely to undergo an expansion in the future as in virtually all AML patients there are markers suitable for NGS analysis. The possibility of discordant dynamics of simultaneous mutations requires comprehensive MRD monitoring combining different techniques (mainly quantitative PCR and NGS). Comprehensive MRD panels targeting all initial markers allows for the detection of dissociated responses of polyclonal disease to therapy with some clones responding while others are refractory. Finally, clinicians should be alert to initiate comprehensive mutation screening in patients with molecular or overt relapse for detecting therapeutic targets such as *IDH1*, *IDH2*, or *FLT3*-ITD/TKD that may newly develop consequently to clonal evolution.

Taken together, hemato-oncologists and pathologists should remain in close interaction with laboratory specialists. An interdisciplinary approach is essential to avoid misinterpretation of results. Both clinicians and molecular biologists require in-depth training for the critical interpretation of NGS results, and their interdisciplinary communication is more essential than ever. Such combined efforts will contribute to an optimized use of these novel diagnostic methods for the benefit of patients with myeloid malignancies.
